# *Akkermansia muciniphila* improves synaptic remodeling and alleviates sepsis-associated cognitive dysfunction *via* regulating the gut-brain axis

**DOI:** 10.3389/fimmu.2026.1925161

**Published:** 2026-10-19

**Authors:** Linxiao Wang, Jiangang Xie, Yan Sun, Yi Zhang, Xi Gao, Yinghui Li, Lin Liu

**Affiliations:** 1 Department of Clinical Laboratory, Honghui Hospital, Xi’an Jiaotong University, Xi’an, Shaanxi, China; 2 Department of Emergency, Honghui Hospital, Xi’an Jiaotong University, Xi’an, Shaanxi, China; 3 Nursing Quality Supervision Office of Key Departments, Honghui Hospital, Xi’an Jiaotong University, Xi’an, Shaanxi, China; 4 Department of Critical Care Medicine, Honghui Hospital, Xi’an Jiaotong University, Xi’an, Shaanxi, China

**Keywords:** *Akkermansia muciniphila*, cognitive dysfunction, gut-brain axis, sepsis, synaptic plasticity

## Abstract

**Background:**

Sepsis-associated cognitive impairment (SACI) is a frequent long-term complication of sepsis characterized by hippocampal neuroinflammation, synaptic loss and gut microbial dysbiosis. *Akkermansia muciniphila* (AKK) possesses anti-inflammatory and intestinal barrier-protective functions, yet its role and mechanism in SACI remain poorly understood.

**Methods:**

In this study, a CLP-induced septic mouse model was used to explore the protective effect of AKK. Behavioral tests were performed to evaluate anxiety and cognitive function. Hippocampal neuronal injury, microglial activation and synaptic plasticity were assessed by HE staining, immunofluorescence, Western blotting and TEM. Intestinal barrier function, gut microbiota composition and metabolic profiles were analyzed *via* 16S rRNA sequencing and untargeted metabolomics, with FMT applied for mechanistic verification.

**Results:**

AKK intervention remarkably alleviated anxiety-like behaviors and rescued cognitive deficits in septic mice. AKK inhibited microglial overactivation, activated the BDNF/P-TrkB pathway, and restored the expression of synaptic SYP and PSD95. Meanwhile, AKK preserved intestinal tight junction integrity, reshaped gut microbiota structure, and reversed sepsis-related metabolic disturbances. FMT further provided evidence that AKK-modulated gut microbiota improved survival and cognitive performance.

**Conclusion:**

In conclusion, AKK ameliorates SACI by suppressing hippocampal neuroinflammation, restoring synaptic plasticity and remodeling the microbiota-gut-brain axis, indicating its potential as a promising candidate for SACI intervention.

## Graphical Abstract

**Figure:**
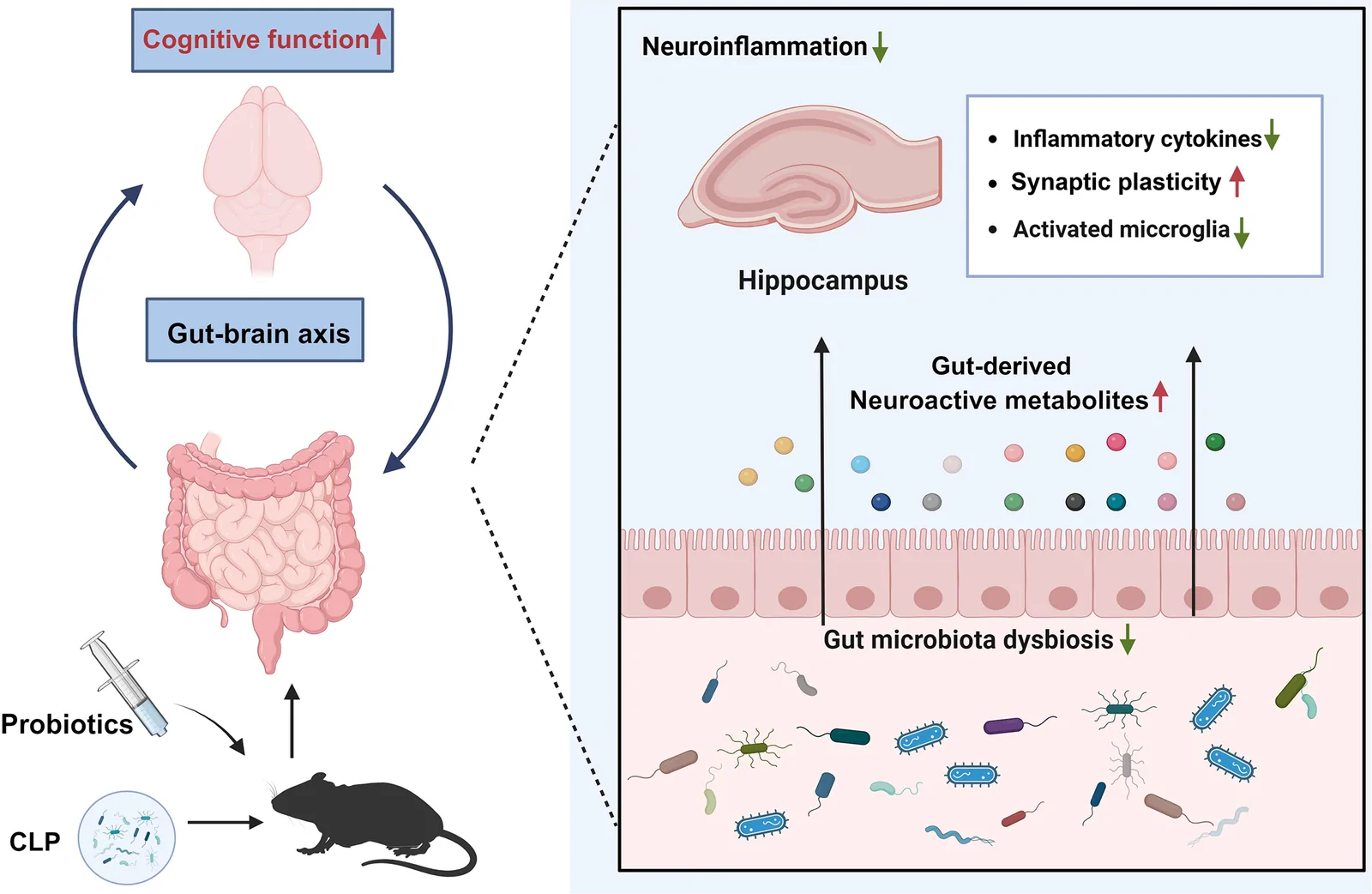

## 1 Introduction

Sepsis is a systemic inflammatory response syndrome triggered by infection, presenting a significant challenge in critical care medicine due to its high morbidity and mortality rates (1). Survivors frequently experience various long-term complications, among which sepsis-associated cognitive impairment (SACI) is particularly common (2). Epidemiological studies reveal that more than 50% of sepsis survivors exhibit notable cognitive deficits, with symptoms persisting for years and, in some cases, leading to permanent disability, severely affecting reintegration into society and overall quality of life (3). SACI is characterized by impaired learning and memory, spatial cognitive deficits, and executive dysfunction, all of which substantially diminish patients’ quality of life and prognosis, potentially resulting in permanent cognitive damage (4). Current clinical interventions for SACI lack specificity and show limited efficacy, largely due to an incomplete understanding of its underlying pathogenesis, hindering the development of targeted therapeutic strategies.

Sepsis induces cognitive dysfunction through central neuroinflammation and hippocampal structural damage, with excessive neuroinflammatory activation being a primary driver of SACI pathogenesis (2). As the resident immune cells of the central nervous system (CNS), microglia are activated by peripheral inflammatory signals during sepsis, releasing large quantities of pro-inflammatory cytokines and establishing a persistent neuroinflammatory environment (5). This cascade results in synaptic pruning, hippocampal neuronal apoptosis, and ultimately cognitive decline (6, 7). Impairments in synaptic plasticity represent a critical structural basis for SACI, with its severity closely linked to disease progression and prognosis (8). Specific mechanisms include microglial M1/M2 polarization imbalance, mitochondrial dysfunction, ferroptosis-mediated lipid peroxidation, and neurotoxicity due to disturbances in tryptophan metabolism (4, 9).

In recent years, advances in microbiome research have highlighted the Microbiota–Gut–Brain Axis (MGBA) as a key area of focus for investigating inflammation-related brain injuries and neurodegenerative diseases, offering new insights into the peripheral regulatory mechanisms of CNS disorders (10, 11). As a central component of the MGBA, the gut microbiota forms a bidirectional regulatory network with the CNS *via* metabolic, immune, neuroendocrine, and vagus nerve pathways (12). During sepsis, significant dysregulation of the gut microbiota occurs, marked by a substantial decrease in species richness and evenness, as well as pathological shifts in community structure (13, 14). However, current research on SACI has largely concentrated on CNS-specific pathological mechanisms, without systematically investigating how gut microbiota dysbiosis regulates hippocampal neuroinflammation, synaptic plasticity, and cognitive function through the MGBA.

*Akkermansia muciniphila* (AKK) is an anaerobic, mucus-degrading commensal that colonizes the outer mucosal layer and is a common member of the healthy gut microbiota (15, 16). By fermenting mucin and supporting epithelial tight junctions, it helps seal the gut barrier, restrains translocation of luminal antigens, and lowers systemic inflammation (12, 17, 18). A growing body of work links AKK to better brain health: it restores cognition in sleep-deprived mice (19), alleviates depressive-like behavior along the gut–liver–brain axis (20), dampens neuroinflammation in Parkinson’s models (21), and reduces postoperative cognitive decline (22). These studies, however, have centered on chronic, progressive conditions. Whether AKK can protect the brain after acute, infection-driven sepsis—and which route it takes to do so—remains unresolved. It is still unclear whether AKK calms overactive hippocampal microglia by restraining their pro-inflammatory M1 polarization, engages the BDNF/TrkB pathway, and rebuilds synapses, or whether these benefits truly act through the gut microbiota rather than a direct systemic effect. This study aims to provide experimental evidence supporting the therapeutic potential of AKK for SACI and to identify promising microbial targets for future SACI interventions.

To test this, we used a CLP-induced sepsis model and included fecal microbiota transplantation (FMT) so that any benefit could be attributed to the microbiota. We asked whether AKK preserves the intestinal barrier and reshapes the fecal microbiota and metabolome; whether it suppresses pro-inflammatory M1 polarization of hippocampal microglia; whether it activates BDNF/P-TrkB and restores SYP and PSD95; and, through these changes, whether it eases anxiety-like behavior and rescues cognition. Our results position AKK as a tractable, microbiota-based candidate for SACI.

## 2 Materials and methods

### 2.1 Experimental animals

SPF-grade male C57BL/6 mice (6–8 weeks, 20–22 g) were obtained from the Experimental Animal Center of Air Force Medical University. Mice were housed under standard conditions (22–25 °C, 50–60% humidity, 12 h light–dark cycle) with free access to food and water. After one-week adaptive feeding, formal experiments were initiated. All animal procedures were approved by the Animal Ethics Committee (IACUC20230038) and conducted in accordance with ethical guidelines to minimize animal distress.

### 2.2 Reagents

*Akkermansia muciniphila* (ATCC BAA-835, Mingzhou Biotechnology Co., Ltd., Ningbo, China) was cultured anaerobically in thioglycollate broth. Cultures were incubated at 37 °C in an anaerobic chamber under a strictly anaerobic N_2_/CO_2_ atmosphere. After incubation to the logarithmic growth phase (24–48 h), bacteria were collected by centrifugation, washed twice with sterile anaerobic PBS, and adjusted to 1 × 10^9^ CFU/mL; the actual dose was verified by serial-dilution plate counting. Primary antibodies were purchased from Abcam (Cambridge, UK), including IBA1 (ab178846), CD68 (ab125212), synaptophysin (ab32166), PSD95 (ab18258), occludin (ab216327), claudin-1 (ab15098), phospho-TrkB (ab75173), BDNF (ab108319), β-actin (ab8227) and GAPDH (ab8245). All secondary antibodies, fixation reagents, protein extraction kits and molecular experimental materials were obtained from commercial suppliers. The chromogenic Limulus Amebocyte Lysate (LAL) endotoxin assay kit (C0276S) was purchased from Beyotime Institute of Biotechnology (Shanghai, China).

### 2.3 Experimental grouping and interventions

#### 2.3.1 AKK intervention experiment

Mice were randomly assigned to four groups (n = 15 per group): Sham, Sham + AKK, CLP, and CLP + AKK. AKK was cultured anaerobically and prepared as a bacterial suspension (1 × 10^9^ CFU/mL). Mice in the Sham + AKK and CLP + AKK groups received daily oral gavage of 0.2 mL of AKK suspension (2 × 10^8^ CFU) (19–21), while the Sham and CLP groups were administered an equivalent volume of sterile PBS for 21 consecutive days. CLP surgery or sham operation was conducted on day 7 of the intervention (**Figure 1A**).

**Figure 1:**
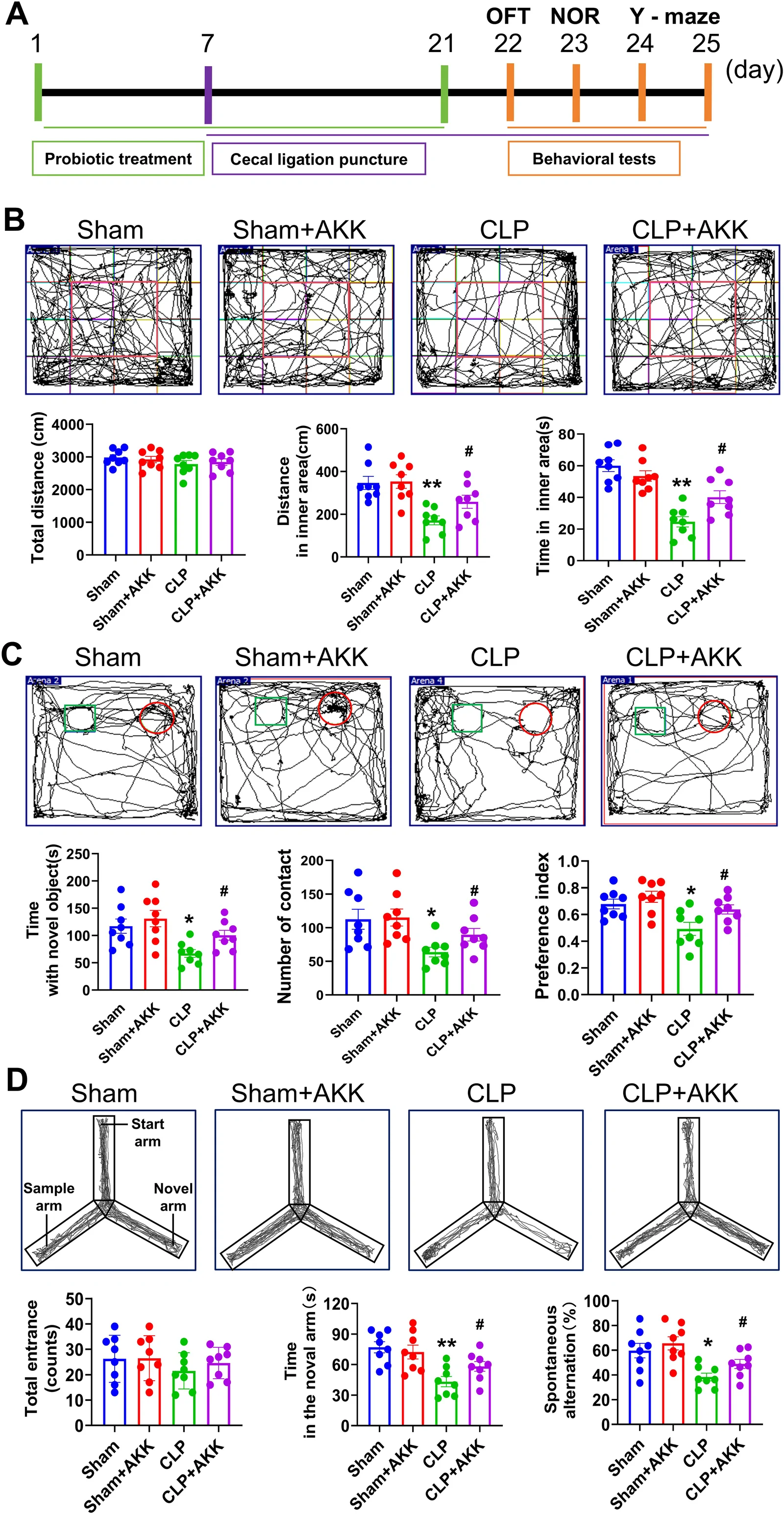
Effects of AKK on behavioral performance in septic mice. **(A)** Experimental timeline, illustrating AKK gavage intervention (2 × 10^8^ CFU/mouse, once daily), CLP modeling, and behavioral testing time points. **(B)** Representative locomotor trajectories in the open field test (OFT) for each group, with statistical analysis of total locomotor distance, central zone distance, and central zone duration (n = 8). **(C)** Representative exploration trajectories in the novel object recognition test (NORT) for each group, with statistical analysis of exploration time within 2 cm of the novel object, contact frequency, and preference index (preference index = novel object exploration time/total exploration time × 100%, n = 8). **(D)** Representative exploration trajectories in the Y-maze test for each group, with statistical analysis of total arm entries, novel arm duration, and spontaneous alternation rate (spontaneous alternation rate = alternation number/[total arm entries – 2] × 100%, n = 8). Sham (sham-operated group); Sham + AKK (sham-operated + AKK intervention group); CLP (sepsis model group); CLP + AKK (sepsis + AKK intervention group). Data are presented as mean ± SEM. *P < 0.05, **P < 0.01 vs. Sham group; ^#^*P <* 0.05 vs. CLP group.

#### 2.3.2 Fecal microbiota transplantation experiment

A separate cohort of mice was divided into three groups (n = 20 per group): CLP + PBS, CLP + trans-CLP, and CLP + trans-AKK. All mice received antibiotic- containing drinking water (ampicillin 1 g/L, vancomycin 0.5 g/L, metronidazole 1 g/L, neomycin 1 g/L) for 5 days to deplete gut microbiota, followed by normal drinking water and fecal microbiota transplantation (FMT) procedures. The CLP + trans-CLP group received fecal microbiota from septic mice, the CLP + trans-AKK group received fecal microbiota from septic mice pretreated with AKK, and the CLP + PBS group was given equivalent PBS.

Preparation of the fecal slurry: fresh fecal pellets were collected from donor mice into sterile pre-weighed tubes and processed immediately (or stored at -80 °C). Feces (200 mg) were suspended in 1 mL sterile pre-reduced PBS and homogenized by vortexing (3000 rpm) until no large particles remained. The suspension was centrifuged at ~800 g for 10 min at 4 °C to remove particulate debris, and the supernatant was passed through a 40–70 µm sterile strainer. The fecal slurry was aliquoted into sterile tubes and kept on ice before use; aliquots were either administered within 12 h or flash-frozen and stored at −80 °C until use. Each recipient received 0.2 mL of the slurry. FMT was performed twice weekly for a total of 3 weeks. CLP surgery was performed one week after the start of FMT, and FMT continued for another 2 weeks post-surgery. Behavioral testing and pathological examination were carried out 2 weeks after CLP surgery (23).

### 2.4 Sepsis model construction

Sepsis was induced by cecal ligation and puncture (CLP). Mice were anesthetized with 1% pentobarbital sodium (50 mg/kg, intraperitoneally). A midline abdominal incision (~1 cm) was made, and the cecum was exposed under sterile conditions. The distal third of the cecum was ligated, and two punctures were made with a 27G needle distal to the ligation site. A small amount of intestinal content was extruded, and the abdominal wall was sutured layer by layer. Sham mice underwent laparotomy without ligation and puncture. All mice received postoperative saline resuscitation (24). At the experimental endpoint, mice were euthanized by intraperitoneal injection of sodium pentobarbital at a dose of 150 mg/kg (3× the anesthetic dose), which induces rapid unconsciousness followed by respiratory and cardiac arrest. Death was confirmed by the absence of corneal reflex, respiratory arrest, and lack of palpable heartbeat.

### 2.5 Behavioral testing

Behavioral testing was performed in quiet, dimly lit, temperature-controlled environments, starting 14 days after model induction. Each group underwent the open field test (OFT), novel object recognition (NOR) test, and Y-maze test sequentially, with 24-hour intervals between each test. Mice were acclimated to the testing environment for 60 minutes prior to each test to minimize environmental stress. Tests were ordered from least to most demanding (OFT, NOR, Y-maze), with a 24-hour interval between tasks. The OFT was run in an empty arena to habituate the mice to the box and to the test room; the NOR subsequently used the same arena, whereas the Y-maze used a separate apparatus. All apparatuses were cleaned with 70% ethanol between animals.

#### 2.5.1 Open field test

In the OFT, mice were gently placed in the center of a 50 × 50 × 30 cm open field arena, and their locomotor trajectories were recorded for 10 minutes. Image analysis software quantified total locomotor distance, central zone distance, and central zone duration. Total distance reflected spontaneous locomotor activity, while central zone parameters indicated anxiety-like behavior (25).

#### 2.5.2 Novel object recognition test

The NOR test consisted of three phases: habituation, familiarization, and testing. During habituation, mice freely explored an empty arena (50 × 50 × 30 cm) for 10 minutes. In the familiarization phase, two identical objects were placed in fixed positions, and exploration time was recorded for 10 minutes. In the testing phase, one object was replaced with a novel object (distinct in shape, color, and material), and exploration of both the novel and familiar objects was recorded for 5 minutes. The preference index was calculated as: (novel object exploration time/total exploration time) × 100%, reflecting recognition memory (19).

#### 2.5.3 Y-maze test

The Y-maze test consisted of three arms (30 × 8 × 15 cm each) arranged at 120° angles. The test comprised acclimation and testing phases. During acclimation, mice freely explored the maze for 5 minutes. For testing, one arm (the novel arm) was blocked, and mice were placed in the start arm for 10 minutes of exploration. After 24 hours, all arms were opened, and mice were placed in the start arm. Total arm entries, novel arm duration, and spontaneous alternation rate were recorded over 5 minutes. The spontaneous alternation rate was calculated as: (alternation number/[total arm entries - 2]) × 100%, reflecting spatial working memory (25).

### 2.6 Quantitative real-time polymerase chain reaction

Total RNA was isolated from hippocampal tissues using commercial extraction kits, and RNA purity was determined *via* a NanoDrop spectrophotometer. Valid RNA was reversely transcribed into cDNA for qPCR detection with SYBR Green reagent. The amplification protocol consisted of an initial denaturation at 95 °C for 3 min, followed by 40 cycles (95 °C for 10 s and 60 °C for 30 s). β-actin served as the internal reference, and relative gene expression was calculated using the 2–ΔΔCt method. All primer sequences are listed in **Supplementary Table S1**.

### 2.7 Hematoxylin-eosin staining

Paraffin-embedded hippocampal section (4 μm thick) were deparaffinized, rehydrated, and stained with hematoxylin and eosin. Neuronal morphology in the DG and CA3 regions was observed under a light microscope, and the arrangement, density, and nuclear integrity of hippocampal neurons were evaluated.

### 2.8 Immunofluorescence staining

After dewaxing and antigen retrieval, sections were incubated with primary antibodies against IBA1, CD68, SYP and PSD95 (1:200–1:300) overnight at 4 °C, followed by fluorescence-conjugated secondary antibodies (1:500) for 1 h at room temperature in the dark; nuclei were counterstained with DAPI. Fluorescence images were captured by confocal microscopy and quantified with ImageJ (26).

### 2.9 Western blotting

Hippocampal and colonic tissues were lysed, and protein concentrations were determined by BCA assay. Proteins were separated by SDS-PAGE and transferred to PVDF membranes. Membranes were incubated with primary antibodies overnight at 4 °C (1:1000; β-actin and GAPDH at 1:5000), followed by HRP-conjugated secondary antibodies (1:5000) for 1 h at room temperature. Protein bands were visualized by ECL and analyzed by ImageJ using GAPDH as an internal control (27).

### 2.10 Plasma endotoxin (LPS) measurement

Orbital blood was collected into endotoxin-free, heparinized tubes and centrifuged at 3000 rpm for 15 min at 4 °C; the plasma was aliquoted and stored at −80 °C. Before assay, plasma was heat-inactivated at 70 °C for 10 min to remove endogenous LAL inhibitors and diluted 1:10 in endotoxin-free water. Endotoxin was measured with a chromogenic LAL kit (Beyotime, C0276S): samples and serially diluted endotoxin standards were incubated with LAL reagent at 37 °C, the chromogenic substrate and stop solution were added, and absorbance was read at 545 nm. Concentrations were calculated from the standard curve, expressed as EU/mL, and all samples were run in duplicate.

### 2.11 Transmission electron microscopy

Hippocampal tissues were fixed in glutaraldehyde and osmium tetroxide, dehydrated and embedded in epoxy resin. Ultrathin sections (50 nm) were stained with uranyl acetate and lead citrate. Synaptic ultrastructure was observed under a transmission electron microscope, and six random fields were selected to count intact synapses for synaptic plasticity assessment (28).

### 2.12 16S rRNA gene sequencing

Total genomic DNA was extracted from fecal samples, and the V3–V4 region of the bacterial 16S rRNA gene was amplified using universal primers 338F and 806R. Purified PCR products were sequenced on the Illumina MiSeq platform. Raw data were quality-filtered and clustered into OTUs at 97% similarity. Alpha diversity (Sobs, Chao1, Shannon) and β-diversity (Bray–Curtis dissimilarity, PCoA) were analyzed to evaluate microbial diversity and structural differences. Differential bacterial taxa were screened by LEfSe (LDA > 4.0), and KEGG analysis was performed for microbial functional prediction (29). All sequencing and bioinformatic analyses were performed by Genedenovo Biotechnology Co., Ltd. (Guangzhou, China).

### 2.13 Untargeted metabolomics analysis

Fecal metabolites were extracted with methanol-water solution, and supernatants were collected after ultrasonic treatment and centrifugation. Samples were analyzed *via* UHPLC-MS/MS for untargeted metabolomics. OPLS-DA was used to distinguish metabolic profiles, and differential metabolites were screened with VIP > 1 and *P <* 0.05. KEGG enrichment analysis was further conducted to explore relevant metabolic pathways. The untargeted metabolomics analysis was performed by Genedenovo Biotechnology Co., Ltd. (Guangzhou, China).

### 2.14 Statistical analysis

All statistical analyses were performed using GraphPad Prism 8.0. The sample sizes were n = 8 for behavioral tests and n = 6 for molecular and histological detections. All data were presented as mean ± SEM. Two-group comparisons were performed using unpaired t-tests, and multi-group differences were analyzed by one-way ANOVA with LSD *post-hoc* test. Survival curves were compared using the Log-rank (Mantel-Cox) test. *P <* 0.05 was considered statistically significant.

## 3 Results

### 3.1 AKK ameliorates cognitive dysfunction in septic mice

To investigate the effects of AKK on cognitive dysfunction in septic mice, behavioral assessments were performed using OFT, NOR test, and Y-maze test. As illustrated in **Figure 1A**, mice underwent probiotic treatment containing AKK for one week, followed by CLP surgery on day 7 to induce sepsis. Behavioral evaluations were conducted between days 21 and 25 to assess cognitive function.

Following CLP, mice showed transient acute morbidity during the first 24–72 h (reduced activity, piloerection, arched back, and reduced feeding), after which the survivors gradually recovered. The 7-day survival rates were 100% in both Sham groups, 53.3% (8/15) in the CLP group, and 66.7% (10/15) in the CLP + AKK group. By day 7–14, the surviving mice had largely recovered and showed no major differences from controls, and behavioral testing was performed from day 14 onward. The detailed post-operative clinical observations are provided in **Supplementary Text S1**.

Behavioral testing results indicated that, compared to the sham-operated group, the CLP group exhibited no significant difference in total locomotor distance but showed significantly reduced central zone distance and duration in OFT, suggesting exacerbated anxiety-like behavior (*P <* 0.05, **Figure 1B**). In the NOR test, exploration time, contact frequency, and the preference index for novel objects were significantly reduced, reflecting impaired recognition memory (*P <* 0.05, **Figure 1C**). In the Y-maze test, while total arm entries showed no intergroup differences, novel arm duration and spontaneous alternation rate were significantly decreased, indicating impaired spatial working memory (*P <* 0.05, **Figure 1D**). Following AKK intervention, all behavioral indices in the CLP + AKK group improved significantly, with alleviated anxiety-like behavior and restored recognition and spatial working memory (*P <* 0.05, **Figures 1B–D**). No statistical differences were observed between the Sham + AKK and Sham groups for any behavioral measures, suggesting that AKK does not adversely affect cognitive function in normal mice.

### 3.2 AKK attenuates intestinal barrier injury and hippocampal pathological damage in septic mice

To assess the effects of AKK on intestinal barrier injury and hippocampal pathological damage in septic mice, histopathological staining and protein expression analysis were performed. Intestinal mucosal HE staining (**Figure 2A**) revealed that, compared to the Sham group, the CLP group exhibited disrupted intestinal villi structures and compromised epithelial integrity. In contrast, the CLP + AKK group showed significant protection of intestinal villi structural integrity, with a marked reduction in sepsis-induced intestinal mucosal damage. Western blot analysis (**Figure 2B**) confirmed that colonic tight junction proteins Occludin and Claudin-1 were significantly downregulated in the CLP group. AKK treatment effectively upregulated the expression of these tight junction proteins, thereby restoring damaged intestinal barrier function. Consistent with the improved tight-junction expression, plasma LPS levels remained low in both Sham and Sham+AKK groups (0.27 ± 0.02 and 0.26 ± 0.04 EU/mL, respectively) but increased markedly in the CLP group (3.75 ± 0.34 EU/mL). AKK treatment significantly reduced plasma LPS in CLP mice (2.49 ± 0.32 EU/mL, **Figure 2C**), indicating that AKK attenuates sepsis-induced intestinal barrier leakage and systemic endotoxin translocation.

**Figure 2:**
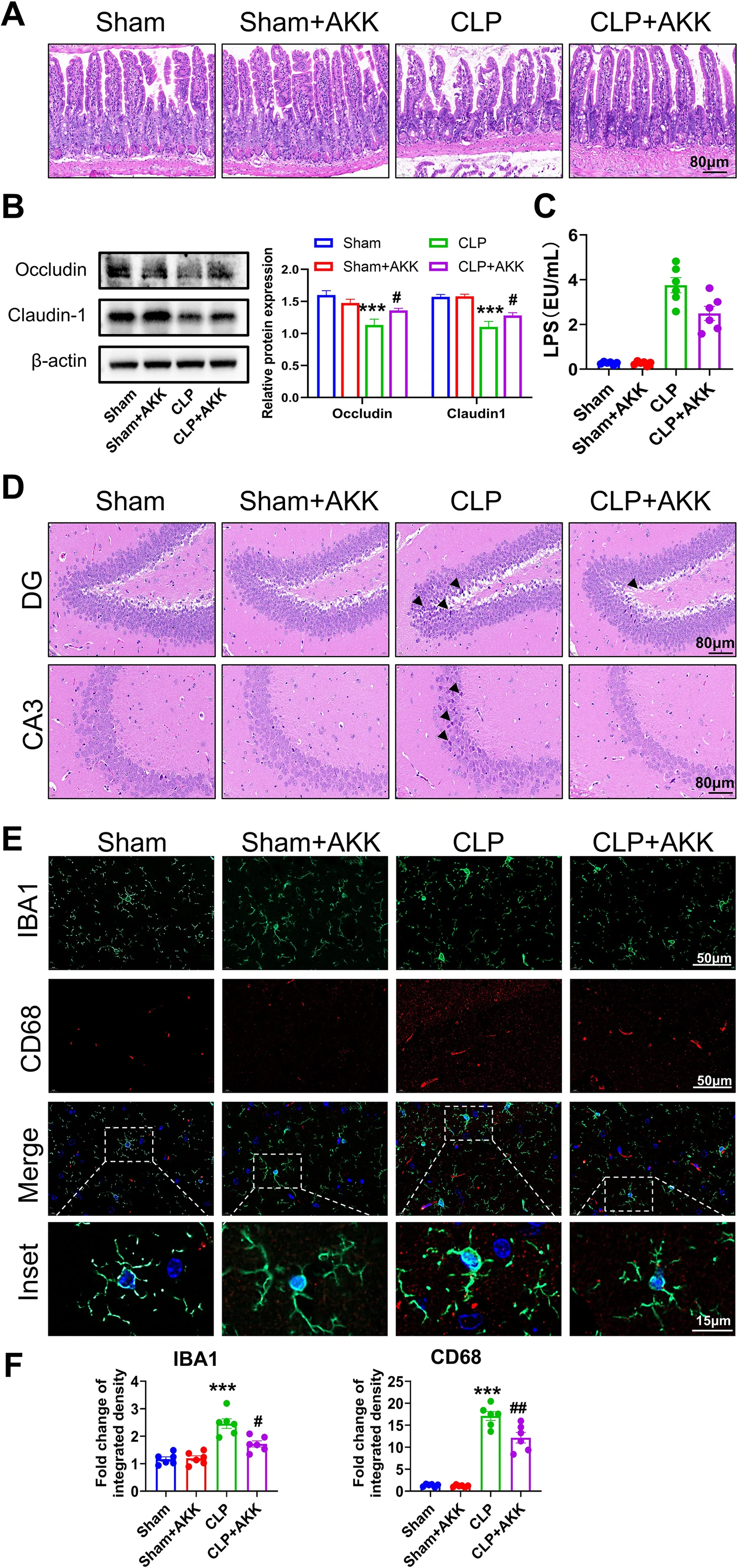
AKK ameliorates intestinal and hippocampal pathology and suppresses neuroinflammation in septic mice. **(A)** Representative H&E staining images of mouse intestinal mucosa (scale bar: 80 μm), demonstrating the structural integrity of intestinal villi (n = 6). **(B)** Left: Western blot bands of colonic tight junction proteins Occludin and Claudin-1, with β-actin as the internal reference; Right: Quantitative analysis of the relative protein expression levels of Occludin and Claudin-1 (n = 6). **(C)** Plasma LPS (endotoxin) levels measured by a chromogenic LAL assay, expressed as EU/mL (n = 6). **(D)** Representative H&E staining images of the hippocampal DG and CA3 regions (scale bar: 80 μm), with arrows highlighting areas of damaged neurons (n = 6). **(E)** Representative immunofluorescence staining images of microglial markers IBA1 (green) and CD68 (red) in the hippocampal CA1 region, with DAPI (blue) labeling cell nuclei (scale bar: 50 μm); the lower panel displays a magnified view of the boxed area (Inset, scale bar: 15 μm, n = 6). **(F)** Quantitative analysis of individual integrated density for IBA1 and CD68 immunofluorescence signals (n = 6). Data are presented as mean ± SEM. Compared to the Sham group: ***P < 0.001; compared to the CLP group: ^#^*P <* 0.05, ^##^*P <* 0.01.

Hippocampal dentate gyrus (DG) and CA3 region HE staining (**Figure 2D**) demonstrated that Sham and Sham + AKK group mice exhibited well-organized, densely packed neurons with normal morphology, uniform intercellular spaces, and no significant inflammatory cell infiltration. The CLP group, however, displayed typical pathological damage, including disorganized, loosely arranged neurons with abnormal morphology, pyknotic nuclei, and markedly widened intercellular spaces. AKK intervention significantly reversed these hippocampal pathological changes. Immunofluorescence staining and statistical analysis (**Figures 2E, F**) showed that microglial markers IBA1 and CD68 were significantly elevated in the hippocampal CA1 region of the CLP group, indicating microglial overactivation (*P <* 0.05). AKK treatment significantly reduced the fluorescence intensity of IBA1 and CD68 (*P <* 0.05), effectively inhibiting sepsis-induced abnormal microglial activation. In the merged images, partial overlap was observed between CD68 signals and IBA1^+^ cell bodies (**Figure 2E**, inset), confirming that the activated cells are microglia.

### 3.3 AKK reverses hippocampal inflammatory response and synaptic plasticity impairment in septic mice

To explore the regulatory effects of AKK on hippocampal inflammatory responses and synaptic plasticity, further molecular biology analyses were conducted. Real-time fluorescence quantitative PCR (**Figure 3A**) revealed that, compared to the Sham group, the CLP group exhibited significantly increased mRNA expression levels of pro-inflammatory cytokines *Il1β* and *Il6*, together with the M1 microglial markers *Aif1*, *Nos2* and *Cd86*, whereas the M2 markers *Arg1* and *Mrc1* and the synapse-associated molecules *Bdnf*, *Syn*, and *Psd95* were significantly downregulated (*P* < 0.05). AKK intervention effectively reversed these abnormal gene expression patterns. Immunofluorescence staining (**Figure 3B**) showed that, compared to the Sham group, fluorescence intensity for the hippocampal presynaptic protein SYP and postsynaptic protein PSD95 was significantly reduced in the CLP group. AKK treatment significantly upregulated both SYP and PSD95 fluorescence intensities, repairing damaged synaptic structures (*P <* 0.05). Merged images showed SYP^+^ and PSD95^+^ puncta in close apposition, confirming their pre- and post-synaptic localization (**Figure 3B**). Western blotting (**Figures 3C, D**) further validated these findings. No significant differences were observed between the Sham + AKK and Sham groups regarding protein expression levels. Compared to the Sham group, the CLP group exhibited significantly reduced expression of BDNF, P-TrkB, SYP, and PSD95, along with significantly increased expression of microglial activation markers IBA1 and CD68 (*P <* 0.05). AKK intervention reversed these abnormal protein expressions, activating the BDNF/P-TrkB signaling pathway, promoting synaptic protein generation, and suppressing microglial activation.

**Figure 3:**
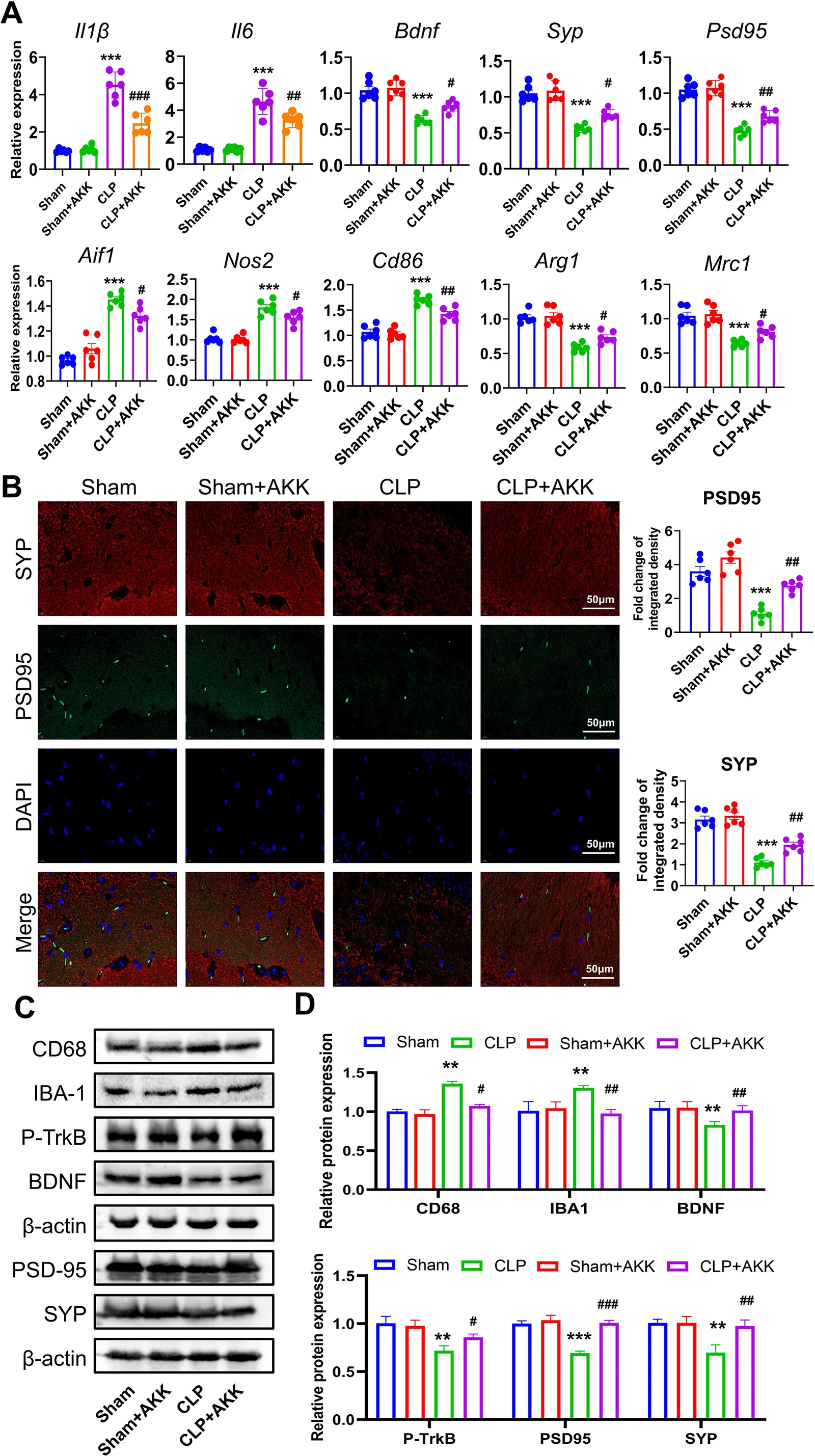
AKK regulates the expression of hippocampal inflammatory factors and synaptic plasticity-associated molecules in septic mice. **(A)** Analysis of relative mRNA expression levels of inflammatory factors *Il1β*, *Il6*, microglial polarization markers (M1: *Aif1*, *Nos2*, *Cd86*; M2: *Arg1*, *Mrc1*), and synaptic-associated genes *Bdnf*, *Syn*, and *Psd95* in hippocampal tissue (n = 6). **(B)** Representative immunofluorescence co-staining images of presynaptic protein SYP (red) and postsynaptic protein PSD95 (green) in the hippocampal CA1 region (scale bar: 50 μm), with quantitative analysis of the integrated density of SYP and PSD95 (n = 6). **(C)** Western blot bands of CD68, IBA1, phosphorylated TrkB (P-TrkB), BDNF, PSD95, and SYP in hippocampal tissue, with β-actin as the internal reference (n = 6). **(D)** Quantitative analysis of the relative protein expression levels corresponding to the Western blot bands above (n = 6). Data are presented as mean ± SEM. Compared to the Sham group: *P < 0.05, **P < 0.01, ***P < 0.001; compared to the CLP group: #*P <* 0.05, ##*P <* 0.01, ###*P <* 0.001.

### 3.4 AKK regulates gut microbiota structure and metabolic profiles in septic mice

To elucidate the mechanisms underlying AKK’s protective effects, 16S rRNA gene sequencing and ultra-high performance liquid chromatography-tandem mass spectrometry (UHPLC-MS/MS) untargeted metabolomics were utilized to assess AKK’s impact on gut microbiota structure and fecal metabolic profiles in septic mice. 16S rRNA sequencing revealed that, compared to the CLP group, the CLP + AKK group exhibited significantly increased gut microbiota α-diversity indices (Sob index, Chao1 index, and Shannon index) (*P <* 0.05, **Figure 4A**), indicating substantial improvements in bacterial richness and diversity. PCoA based on Bray-Curtis distance demonstrated significant structural differences in microbiota between groups, with clear sample clustering and high discrimination (**Figure 4B**). Venn diagrams further highlighted genus-level differences between the groups (**Figure 4C**). The CLP + AKK group showed a significant increase in the abundance of beneficial bacteria, including *Akkermansia* and *Rikenellaceae*, while the abundance of pathogenic bacteria, such as *Enterobacteriaceae* and *Escherichia-Shigella*, was significantly reduced (*P <* 0.05, **Figures 4D, E**). KEGG pathway prediction analysis indicated significant functional differences across groups in key pathways, including amino acid metabolism, lipid metabolism, and immune regulation (**Figure 4F**).

**Figure 4:**
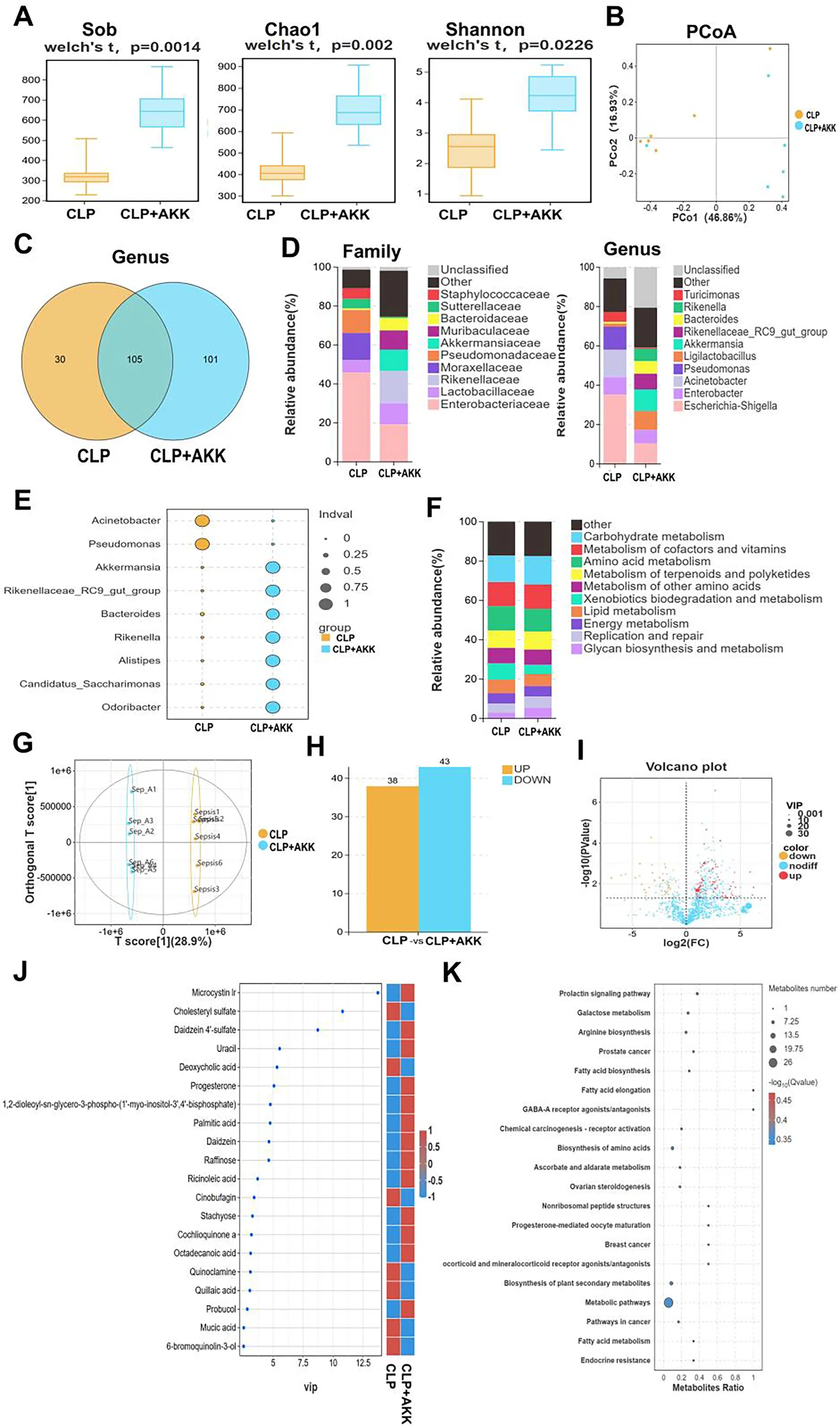
Gut microbiota and metabolomic analysis in septic mice. **(A)** Box plots of gut microbiota α-diversity indices, including Sob, Chao1, and Shannon indices. **(B)** Principal coordinate analysis (PCoA) plot of gut microbiota β-diversity based on Bray-Curtis distance. **(C)** Venn diagram at the genus level. **(D)** Relative abundance bar chart at the family level. **(E)** LEfSe analysis plot showing significantly different bacterial genera between groups. **(F)** KEGG pathway functional prediction bar chart (Top 10 pathways). **(G)** OPLS-DA score plot of fecal metabolomics. **(H)** Volcano plot of differential metabolites (VIP > 1 and *P <* 0.05). **(I)** Hierarchical clustering heatmap of differential metabolites. **(J)** KEGG pathway enrichment analysis bubble plot of differential metabolites. **(K)** Bubble plot for KEGG pathway enrichment analysis of altered metabolites. n = 6.

To further explore the metabolic mechanisms by which AKK modulates the gut–brain axis, UHPLC-MS/MS untargeted metabolomics was employed to examine fecal metabolic profile differences. OPLS-DA score plots revealed clear sample clustering and significant separation among groups, suggesting that both sepsis modeling and AKK intervention notably altered the overall metabolic profile (**Figure 4G**). Based on the screening criteria of VIP > 1 and *P <* 0.05, a total of 81 differential metabolites were identified. Volcano plot and heatmap visualizations revealed that 43 metabolites were downregulated and 38 metabolites were upregulated in the CLP group (**Figures 4H, I**). VIP plots displayed the top 20 differential metabolites and their respective expression heatmaps, showing that Uracil, Daidzein 4’-sulfate, and Palmitic acid were significantly elevated following AKK treatment, while Cholesteryl sulfate was highly expressed in the sepsis group (**Figure 4J**). KEGG pathway enrichment analysis indicated that metabolic pathways were the most significantly enriched, containing the largest number of differential metabolites, which suggests extensive metabolic disruption in the sepsis state. Additionally, fatty acid metabolism and cancer-related pathways also showed significant enrichment. The enrichment was dominated by pathways linked to amino-acid, fatty-acid, and bile-acid metabolism, which are directly relevant to neuroinflammation and synaptic function. A few broad KEGG categories (e.g., cancer-related and prolactin signaling) also appeared as secondary hits (**Figure 4K**); as nonspecific terms reflecting the generalized metabolic disturbance of sepsis, they are not pursued further here.

### 3.5 AKK-mediated fecal microbiota transplantation improves survival rate and alleviates cognitive dysfunction in septic mice

To verify the direct protective effects of AKK-mediated gut microbiota restructuring on cognitive dysfunction in septic mice, FMT experiments were performed, followed by statistical analysis of survival rates, body weight changes, behavioral testing, and histopathological staining. As shown in **Figure 5A**, mice first received antibiotic treatment to deplete their indigenous gut microbiota, followed by FMT, CLP surgery to establish the sepsis model, and subsequent behavioral and histological assessments.

**Figure 5:**
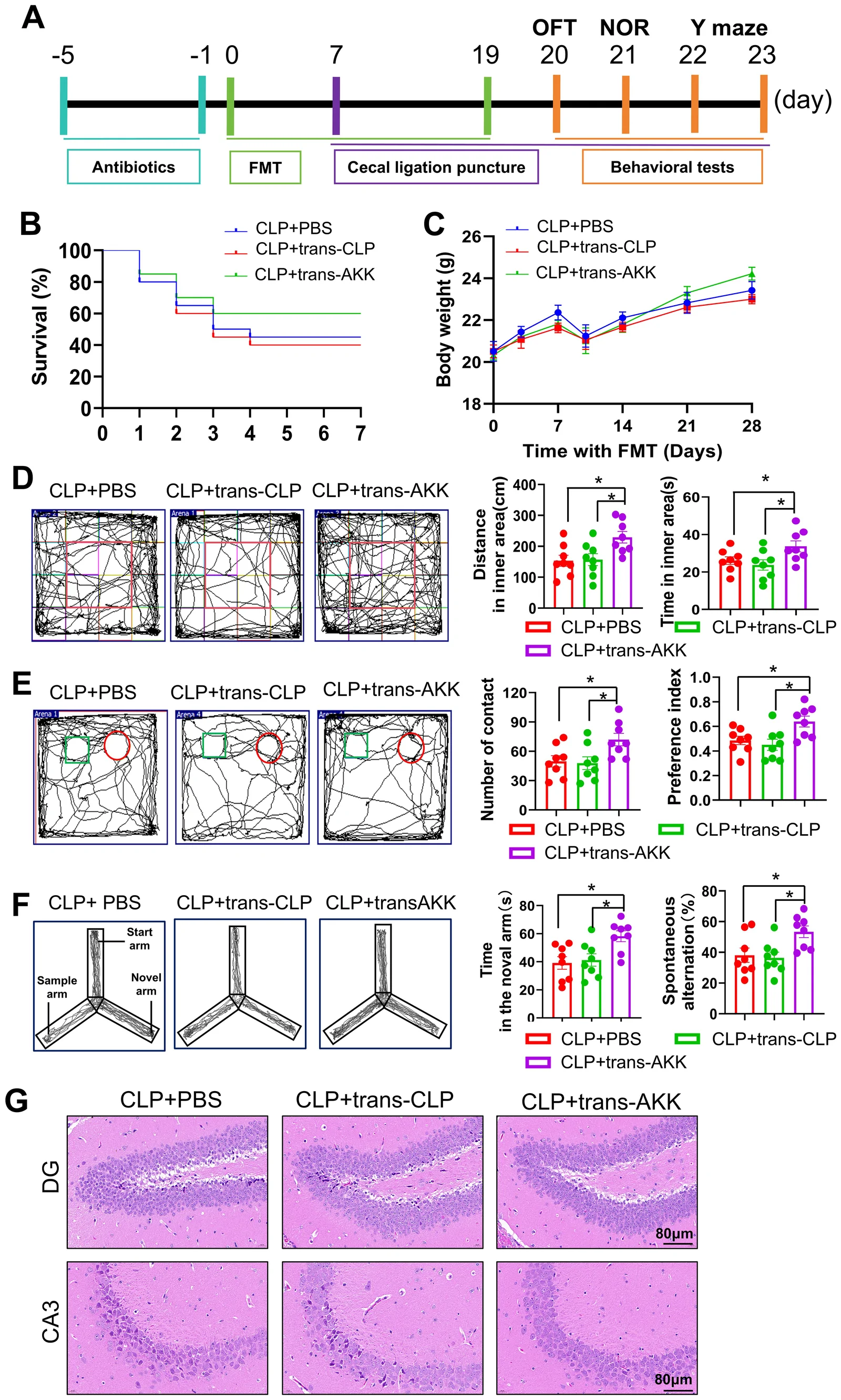
Effects of fecal microbiota transplantation on cognitive impairment in septic mice. **(A)** Schematic diagram of FMT experimental timeline, showing antibiotic pretreatment, fecal microbiota transplantation, CLP modeling, and behavioral testing time points. **(B)** Kaplan-Meier survival curve for 7-day survival rate (n = 20). **(C)** Body weight changes in CLP mice following FMT (n = 8). **(D)** Representative locomotor trajectories in the open field test and statistical analysis of central zone distance and central zone duration (n = 8). **(E)** Representative exploration trajectories in the novel object recognition test and statistical analysis of novel object preference index (n = 8). **(F)** Representative exploration trajectories in the Y-maze test and statistical analysis of novel arm duration and spontaneous alternation rate (n = 8). **(G)** H&E staining sections of hippocampal DG and CA3 regions (scale bar = 80 μm, n = 6). CLP + PBS (sepsis + PBS gavage group); CLP + trans-CLP (sepsis + fecal microbiota transplantation from septic mice group); CLP + trans-AKK (sepsis + fecal microbiota transplantation from AKK-treated septic mice group). *P < 0.05.

Behavioral observations revealed that following CLP surgery, nesting behavior was suppressed, body temperature dropped, and some mice experienced early mortality due to infection. Kaplan–Meier survival analysis showed that mortality in CLP mice mainly occurred within the first 7 days, with most surviving mice living until day 28 after the critical period. Therefore, the 7-day survival rate was analyzed. The 7-day survival rates were 45%, 40%, and 60% in the CLP + PBS, CLP + trans-CLP, and CLP + trans-AKK groups, respectively, with the CLP + trans-AKK group exhibiting a significantly higher 7-day survival rate than the other two groups (*P <* 0.05, **Figure 5B**). Following CLP surgery, food and water intake significantly decreased, and body weight dropped sharply in the short term. Long-term observation revealed that AKK FMT effectively alleviated body weight decline in septic mice (**Figure 5C**). Behavioral testing results showed that compared to the CLP + PBS and CLP + trans-CLP groups, the CLP + trans-AKK group exhibited significantly improved central zone activity in OFT, indicating reduced anxiety-like behavior (*P <* 0.05, **Figure 5D**). Furthermore, the novel object preference index was significantly elevated, suggesting enhanced recognition memory (*P <* 0.05, **Figure 5E**), and the spontaneous alternation rate in the Y-maze was significantly increased, indicating restored spatial working memory (*P <* 0.05, **Figure 5F**). HE staining revealed that pathological damage in the hippocampal DG and CA3 regions was significantly attenuated in the CLP + trans-AKK group, with more regularly arranged neurons and reduced inflammatory infiltration (**Figure 5G**), indicating that AKK-mediated gut microbiota restructuring confers protective effects against sepsis-associated cognitive dysfunction.

### 3.6 AKK-enriched fecal microbiota transplantation restores hippocampal synaptic plasticity by suppressing microglial activation

To determine whether FMT improves hippocampal synaptic plasticity in septic mice through AKK transmission, immunofluorescence staining, TEM, and Western blotting were performed. Immunofluorescence staining and integrated optical density analysis revealed that, compared to the CLP + PBS and CLP + trans-CLP groups, hippocampal SYP and PSD95 fluorescence intensities were significantly higher in the CLP + trans-AKK group (*P <* 0.05, **Figures 6A, B**), indicating enhanced synapse-associated protein expression.

**Figure 6:**
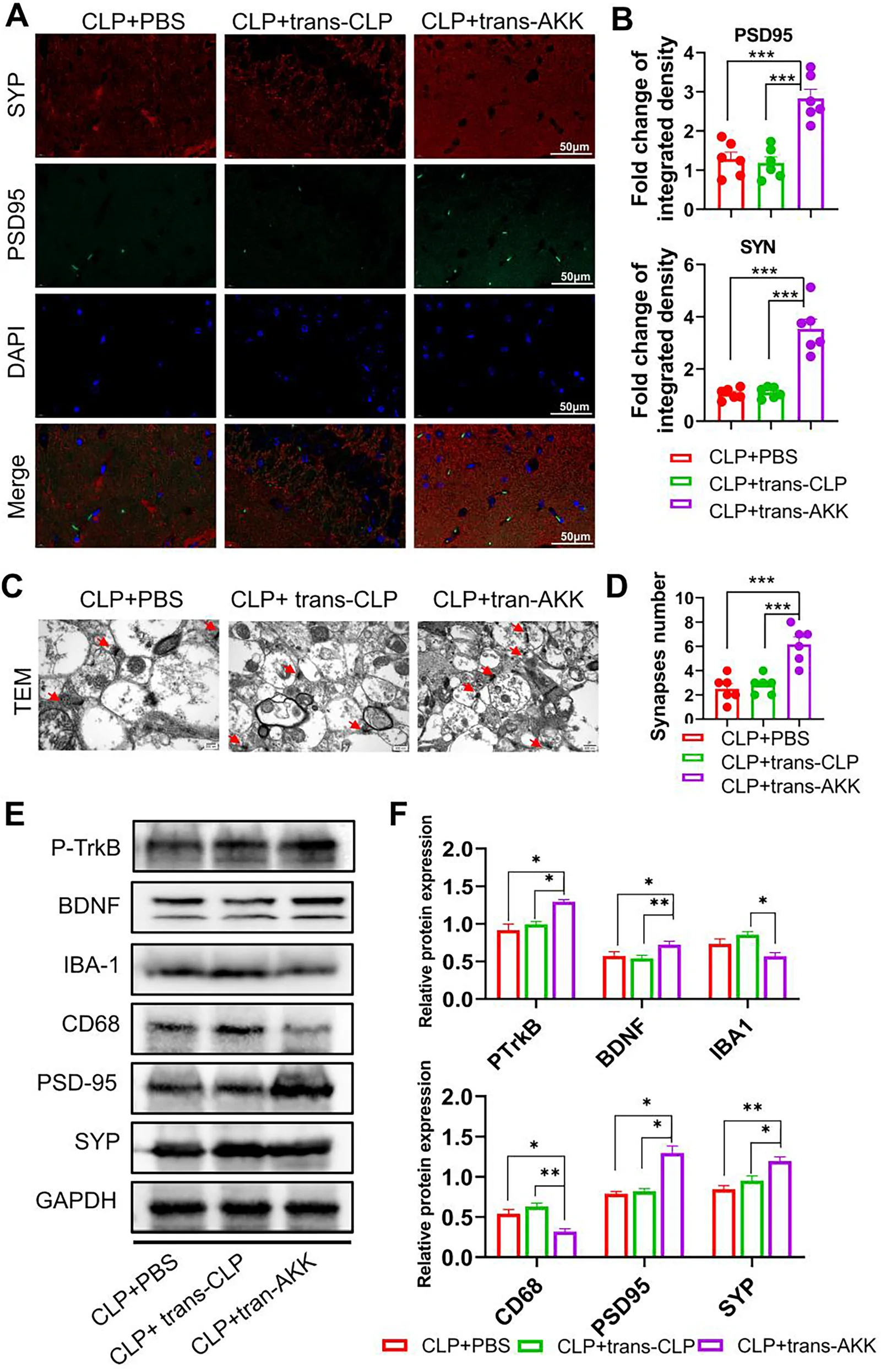
Effects of fecal microbiota transplantation on hippocampal synaptic plasticity in septic mice. **(A)** Immunofluorescence staining images and merged images of hippocampal synapse-associated markers SYP and PSD95 (scale bar = 50 μm, n = 6). **(B)** Quantitative statistical analysis of integrated density for hippocampal SYP and PSD95 immunofluorescence (n = 6). **(C)** Transmission electron microscopy (TEM) images of hippocampal synapses (scale bar = 500 nm), with red arrows indicating intact synaptic structures. **(D)** Statistical analysis of hippocampal synapse numbers (n = 6). **(E)** Western blot detection bands of hippocampal proteins, including P-TrkB, BDNF, IBA1, CD68, PSD95 and SYP, with GAPDH as internal control (n = 6). **(F)** Statistical analysis of relative expression of hippocampal P-TrkB, BDNF, IBA1, CD68, SYP, and PSD95 proteins (n = 6). *P < 0.05, **P < 0.01, ***P < 0.001.

TEM observations and quantitative analysis showed that the CLP + PBS and CLP + trans-CLP groups exhibited significantly reduced hippocampal synapse numbers and severe ultrastructural damage (**Figure 6C**). In contrast, the CLP + trans-AKK group displayed a significant increase in synapse number (*P <* 0.05, **Figures 6C, D**) and near-normalized ultrastructure. Western blotting further confirmed that hippocampal SYP and PSD95 expression were significantly elevated, while IBA1 and CD68 levels were significantly reduced in the CLP + trans-AKK group (*P <* 0.05, **Figures 6E, F**). Notably, the BDNF/P-TrkB pathway was also recapitulated in the FMT cohort: both BDNF and P-TrkB protein levels were significantly higher in the CLP + trans-AKK group than in the CLP + PBS and CLP + trans-CLP groups (*P* < 0.05, **Figures 6E, F**), indicating that AKK-enriched gut microbiota is sufficient to drive BDNF/P-TrkB activation. These findings suggest that FMT effectively transmits AKK-enriched beneficial bacteria and that the metabolites produced after colonization may modulate hippocampal microglial activation, thereby improving hippocampal synaptic plasticity in septic mice.

## 4 Discussion

This study thoroughly examined the role of AKK in ameliorating sepsis-associated cognitive dysfunction and its underlying mechanisms. It revealed AKK’s multifaceted beneficial effects on cognitive function, neural injury, synaptic plasticity, intestinal barrier integrity, and microbiota composition in CLP-induced septic mice, and FMT provided independent support that AKK-mediated gut microbiota remodeling contributes to the improvement of sepsis-associated cognitive dysfunction.

Extensive research has consistently demonstrated that cognitive impairment is prevalent in sepsis mouse models, aligning closely with the clinical manifestations of human sepsis-associated encephalopathy (SAE) (30, 31). In the present study, AKK intervention remarkably relieved anxiety-like behaviors, and rescued recognition and spatial working memory in septic mice. These findings align with AKK’s neuroprotective effects observed in other neurological disease models. For instance, AKK has been shown to mitigate sleep deprivation-induced cognitive impairment in mice and regulate microglial synaptic pruning (19). Furthermore, AKK has demonstrated effectiveness in improving depressive-like symptoms in alcohol-LPS-induced mouse models by modulating 5-HT neurotransmitter levels (32, 33). In PD models, AKK alleviates neuroinflammation and promotes neurogenesis (21). These studies highlight AKK’s potential in improving cognitive dysfunction and neuropsychiatric symptoms across a range of pathological conditions.

Sepsis-induced intestinal barrier dysfunction is a key contributor to the systemic inflammatory response and distal organ injury (34). Our study demonstrated that CLP mice exhibited disrupted intestinal villi, compromised epithelial integrity, and significantly reduced expression of the tight junction proteins Occludin and Claudin-1. AKK intervention effectively repaired the damaged intestinal barrier. Intestinal barrier integrity is essential for preventing bacterial translocation and toxin entry into the bloodstream, and its restoration can significantly reduce systemic inflammation, mitigating its impact on the CNS (35). In the hippocampus, CLP induced neuronal disarrangement and obvious microglial overactivation (elevated IBA1/CD68 expression). AKK markedly inhibited microglial hyperactivation and neuroinflammatory cascade, which is critical for preventing neuronal damage and cognitive decline. Notably, AKK suppressed M1-associated genes (*Aif1*, *Nos2*, *Cd86*, *Il1β* and *Il6*) while restoring M2-associated genes (*Arg1* and *Mrc1*), indicating that AKK does not merely reduce microglial number but suppresses the pro-inflammatory M1 program. Microglial overactivation, a hallmark of neuroinflammation, leads to the release of pro-inflammatory mediators that damage neurons and impair cognitive function (36, 37). Microglia continuously monitor the neural microenvironment under healthy conditions, clearing metabolic waste and synapses that are no longer functional to maintain CNS homeostasis (38).

We also observed convergent protective phenotypes across distinct *in-vivo* disease models investigating AKK-mediated gut-brain axis regulation. Wang et al. demonstrated that AKK improved cognitive performance and mitigated neuroinflammation in APP/PS1 mice partly through AhR/NF-κB/NLRP3-dependent signaling accompanied by reshaped tryptophan and short-chain fatty-acid metabolism (39). In a chronic metabolic cognitive-impairment model, Du et al. further reported that AKK alleviated diabetic cognitive impairment by suppressing NLRP3 inflammasome activation and rescuing hippocampal synaptic defects (40). Although intestinal-barrier preservation, neuroinflammation suppression and synaptic restoration are shared beneficial outcomes across these studies, disease etiologies differ substantially: chronic neurodegeneration and chronic metabolic disturbance, in contrast to the acute polymicrobial infection-driven SACI in our CLP model. Similar phenotypic improvements do not necessarily indicate identical upstream mechanistic pathways. Potential molecular mediators that could transmit AKK-derived protective signals have also been reported. A study by Gao et al. showed that AKK ameliorates intestinal barrier damage and microglial activation in ischemia–reperfusion (I/R)-induced postoperative cognitive dysfunction (POCD) through its extracellular vesicles (EVs), highlighting AKK’s critical role in modulating host immune responses (22).

Mechanistically, AKK reversed the abnormal expression of hippocampal pro-inflammatory cytokines (*Il1β*, *Il6*) and synaptic-related genes (*Bdnf*, *Syp*, *Psd95*). In models of liver injury-induced cognitive impairment, AKK has been shown to improve cognitive function by regulating BDNF and serotonin pathways within the gut–liver–brain axis (20). SYP and PSD95 are key markers of presynaptic and postsynaptic structures, respectively, with changes in their expression reflecting synaptic integrity and functionality. In AD models, AKK has been found to significantly upregulate hippocampal SYP and PSD95 levels (41). Furthermore, BDNF binds its receptor TrkB and drives its phosphorylation, which in turn engages PI3K/Akt and ERK–CREB signaling that supports neuronal survival and the production of synaptic proteins (42). In our data, BDNF and phospho-TrkB dropped together with SYP and PSD95 after CLP and were brought back by AKK in the same direction—after both direct gavage (**Figures 3C–F**) and FMT (**Figures 6E, F**). We therefore read BDNF–TrkB as a downstream link that turns reduced neuroinflammation and a restored barrier into synaptic and cognitive recovery, rather than the step at which AKK acts directly. These findings demonstrate that AKK improves cognitive dysfunction by suppressing hippocampal inflammation and enhancing synaptic plasticity. This mechanism is consistent with AKK’s anti-inflammatory and neurotrophic effects observed in other neurological diseases (15). Moreover, AKK remodeled gut microbiota composition, increased microbial α-diversity, enriched beneficial taxa including *Akkermansia* and *Rikenellaceae*, and reduced opportunistic pathogens. This composition shift is itself informative: enrichment of *Akkermansia* fits its known barrier-strengthening role, the rise in Rikenellaceae is consistent with anti-inflammatory/SCFA-associated homeostasis, and the reduction of *Enterobacteriaceae* and *Escherichia-Shigella* cuts a major source of gut-derived LPS—agreeing with the lower plasma LPS we observed.

At the metabolic level, untargeted metabolomics showed that AKK reversed sepsis-associated changes in amino-acid, fatty-acid, and bile-acid metabolism, categories previously linked to neuroinflammation and synaptic function. Dysbiosis and metabolic disturbance are hallmarks of sepsis-induced cognitive dysfunction, and these metabolites are likely mediators of AKK’s anti-inflammatory and synaptic-repair effects. For instance, SCFAs produced by the gut microbiota are key signaling molecules of the gut–brain axis (43), and increased *Akkermansia* has been linked to improved motor function and dopaminergic protection in PD models (21, 44). Consistent with previous AD-model observations, these metabolic shifts may contribute to AKK-mediated neuroprotection, yet such effects remain to be validated in acute sepsis settings. One result at first looks contradictory: palmitic acid rose in the AKK group although palmitate can activate TLR4 (45). These are, however, fecal metabolites, so the raised palmitic acid reflects luminal contents rather than the circulating pool reaching the brain. In the same mice, the functional inflammatory markers all fell—plasma LPS (**Figure 2C**), hippocampal IBA1/CD68—while BDNF/TrkB and synaptic proteins rose, so the measured net effect was anti-inflammatory. Whether fecal palmitate has systemic relevance would require targeted measurement of circulating palmitate and TLR4 signaling.

FMT provided independent, microbiome-level evidence that AKK-remodeled microbiota improved survival and alleviated body-weight loss as well as cognitive performance in septic mice, and alleviated hippocampal neuronal injury. One caveat is that all FMT recipients first received broad-spectrum antibiotics, which are themselves therapeutic in CLP and likely compress the survival differences. The similar outcomes of the PBS and trans-CLP groups therefore do not indicate that the microbiota is inert—both remain depleted and neither supplies *Akkermansia*; the benefit lies specifically with the trans-AKK group. Mechanistically, the CLP + trans-AKK group showed elevated hippocampal SYP and PSD95, more synapses, near-normal synaptic ultrastructure, and reduced IBA1/CD68, suggesting that the AKK-enriched microbiota transmitted by FMT curtails hippocampal microglial activation and thereby improves synaptic plasticity. *Akkermansia* supports host health by strengthening the intestinal barrier; in a microbiome-depletion model it activated Wnt/β-catenin signaling to drive intestinal stem-cell proliferation (18), and live AKK releases the RKH tripeptide that binds TLR4 and protects against sepsis-induced organ damage (46). These results also align with recent work showing that AKK upregulates hippocampal decorin, promoting autophagy and suppressing neuronal apoptosis (47).

Several limitations should be acknowledged. We used only the CLP model, which reproduces polymicrobial sepsis but not every feature of SACI, and all experiments were in male mice, so extension to females and other sepsis models remains to be shown. FMT tells us the effect is microbiome-dependent, but we did not isolate a single causal metabolite or strain, and untargeted metabolomics could not assign one functional molecule. The BDNF–TrkB and tight-junction changes are also correlational: we did not test them causally, for example by blocking TrkB, and we judged BBB integrity from plasma LPS rather than a tracer-based permeability assay.

There are translational caveats too. Live AKK is not straightforward in severe sepsis—critically ill patients are usually on broad-spectrum antibiotics, which would suppress the very bacterium given; immunocompromised, septic patients carry a theoretical translocation risk; and delayed gastric emptying and poor absorption make oral dosing unreliable. On top of that, we started AKK a week before CLP and kept it up, so the design is essentially preventive, whereas patients arrive only after sepsis has declared itself and a treatment has to work then. What we show is that early AKK limits the injury; we do not show that it reverses what is already established. The results should therefore be read as preclinical proof-of-concept. Pasteurized AKK or defined bacterial components may avoid the antibiotic and infection concerns, and a therapeutic arm—starting AKK only after CLP—along with dosing, timing, and patient selection, will need dedicated clinical study.

## 5 Conclusion

AKK supplementation ameliorates sepsis-associated anxiety and cognitive dysfunction *via* protecting intestinal barrier integrity, inhibiting hippocampal microglial activation and neuroinflammation, restoring synaptic plasticity, and remodeling gut microbiota and metabolic profiles. FMT suggests that the protective effects of AKK rely on the regulation of the microbiota-gut-brain axis. AKK is expected to be a promising probiotic intervention strategy for SACI.

## Data Availability

The datasets presented in this study can be found in online repositories. The names of the repository/repositories and accession number(s) can be found below: https://www.ncbi.nlm.nih.gov/, PRJNA1417935.
