# Supplementary material for: Akkermansia muciniphila improves synaptic remodeling and alleviates sepsis-associated cognitive dysfunction via regulating the gut-brain axis
Source: Front Immunol. 2026 Oct 19;17:1925161. doi: 10.3389/fimmu.2026.1925161 (PMC13724486; doi:10.3389/fimmu.2026.1925161)
Supplement: Supplementary file 1 [file DataSheet1.docx]

**Supplementary Explanation for Raw Western Blot Images**

Weak visibility of membrane edges and molecular weight markers in some full-length blots results from overexposure during luminescence detection. The housekeeping proteins GAPDH and β-Actin exhibit extremely strong signals, which increased the overall brightness of the membrane and faded peripheral blank regions and marker bands. However, all blots retain at least one empty lane on both sides of the target bands, confirming that no excessive cropping was conducted. All raw images are original and unaltered, and the cropped regions used in the main manuscript are marked with dashed rectangles for easy identification.

**Figure 2B**

**Figure 2B: Uncropped raw WB images.**

Mice were randomly assigned to four groups : Sham, Sham + AKK, CLP, and CLP + AKK. AKK was cultured anaerobically and prepared as a bacterial suspension (1 × 10^9^ CFU/mL). Mice in the Sham + AKK and CLP + AKK groups received daily oral gavage of 0.2 mL of AKK suspension (2 × 10^8^ CFU), while the Sham and CLP groups were administered an equivalent volume of sterile PBS for 21 consecutive days. CLP surgery or sham operation was conducted on day 7 of the intervention (Figure 1A).

**Figure 3C**

**Figure 3C: Uncropped raw WB images**

Mice were randomly assigned to four groups : Sham, Sham + AKK, CLP, and CLP + AKK. AKK was cultured anaerobically and prepared as a bacterial suspension (1 × 10^9^ CFU/mL). Mice in the Sham + AKK and CLP + AKK groups received daily oral gavage of 0.2 mL of AKK suspension (2 × 10^8^ CFU), while the Sham and CLP groups were administered an equivalent volume of sterile PBS for 21 consecutive days. CLP surgery or sham operation was conducted on day 7 of the intervention (Figure 1A).

**Figure 6E**

**Figure 6E: Uncropped raw WB images.**

A separate cohort of mice was divided into three groups : CLP + PBS, CLP + trans-CLP, and CLP + trans-AKK. All mice received antibiotic- containing drinking water (ampicillin 1 g/L, vancomycin 0.5 g/L, metronidazole 1 g/L, neomycin 1 g/L) for 5 days to deplete gut microbiota, followed by normal drinking water and fecal microbiota transplantation (FMT) procedures. The CLP + trans-CLP group received fecal microbiota from septic mice, the CLP + trans-AKK group received fecal microbiota from septic mice pretreated with AKK, and the CLP + PBS group was given equivalent PBS. FMT was performed twice weekly for a total of 3 weeks. CLP surgery was performed one week after the start of FMT, and FMT continued for another 2 weeks post-surgery.
