# Supplementary material for: Akkermansia muciniphila improves synaptic remodeling and alleviates sepsis-associated cognitive dysfunction via regulating the gut-brain axis
Source: Front Immunol. 2026 Oct 19;17:1925161. doi: 10.3389/fimmu.2026.1925161 (PMC13724486; doi:10.3389/fimmu.2026.1925161)
Supplement: Supplementary file 2 [file DataSheet2.docx]

**Supplementary Text S1. Detailed post-operative clinical observations.**

During the acute phase after CLP (0-24 h), septic mice displayed prominent morbidity, including markedly reduced spontaneous activity, absent exploratory behavior, huddling and curling, and sluggish responses to external stimuli. Typical manifestations included piloerection, eyelid closure, increased ocular secretions, arched-back posture, and rapid, shallow breathing.

On post-operative days 2-3, surviving mice showed minimal autonomous activity, occasional feeding, and a mild improvement in mental state and coat condition, with their general status stabilizing. By days 4-7, activity, alertness, feeding, and coat condition gradually recovered, and breathing difficulty largely resolved, although the mice still showed reduced activity, diminished exploration, and slower recovery relative to sham-operated animals.

The 7-day survival rates post-CLP were 100% in the Sham and Sham + AKK groups, 53.3% (8 of 15) in the CLP group, and 66.7% (10 of 15) in the CLP + AKK group. By post-operative days 7–14, ambulation, feeding, breathing, and coat color showed no obvious differences from healthy controls, and survival stabilized. Despite this apparent recovery, the surviving mice still exhibited persistent long-term behavioral sequelae, indicating that sepsis induces sustained neurobehavioral and cognitive deficits.
