# Supplementary material for: Akkermansia muciniphila improves synaptic remodeling and alleviates sepsis-associated cognitive dysfunction via regulating the gut-brain axis
Source: Front Immunol. 2026 Oct 19;17:1925161. doi: 10.3389/fimmu.2026.1925161 (PMC13724486; doi:10.3389/fimmu.2026.1925161)
Supplement: Supplementary file 3 [file Table1.docx]

**Supplementary Table S1. Primer sequences used for qPCR**

| Gene | Forward primer (5'→3') | Reverse primer (5'→3') |
| --- | --- | --- |
| β-actin | AGGCGAACCTCTCAAACACC | GGCCATCTCCTGCTCGAAGTC |
| Il1β | TGCAAGCTTCCTGGTGTGTG | GCTCTGCTTGGGATCCATCTG |
| Il6 | CCTGCAAGAGACTTCCATCCAG | AGTGGTATAGACAGGGTCTTAG |
| Bdnf | CGCAAACGTGGACAAGTGCGA | GGCTTGTTTCTCCCCTCTTCACTT |
| Syn | GGATGGCTTCTACCCCAATG | TCCACACGCTTCTCATCCTC |
| Psd95 | GCTGAGGAGGATGAGGAGGAG | CGTAGACACCGTCACAAAGCA |
| Nos2 | GGCAGCCTGTGAGACCTTTG | TGATTCAATCCCTGAATTGCC |
| Arg1 | CTCCAAGCCAAAGTCCTTAGAG | AGGGAGCTGTCATTAGGGACATC |
| Mrc1 | CTCTGTTCAGCTATTGGACGC | CGGAATTTCTGGGATTCAGCTTC |
| Cd86 | ACGATGGACCCCAGATGCACCA | GCGTCTCCACGGAAACAGCA |
| Aif1 | TCTGCCGTCCAAACTTGAAGCC | CTCTTCAGCTCTAGGTGGGTCT` |

**Supplementary Table S2. Number of mice per group in each experiment.**

Direct AKK intervention experiment (4 groups, n = 15 enrolled per group):

| Group | Enrolled | Survived to day 7 | Behavior (OFT/NOR/Y-maze) | Terminal assays (HE/IF/WB/qPCR) |
| --- | --- | --- | --- | --- |
| Sham | 15 | 15 | 8 | 6 |
| Sham + AKK | 15 | 15 | 8 | 6 |
| CLP | 15 | 8 (53.3%) | 8 | 6 |
| CLP + AKK | 15 | 10 (66.7%) | 8 | 6 |

FMT experiment (3 groups, n = 20 enrolled per group):

| Group | Enrolled | Survived to day 7 | Behavior/body weight | Terminal assays |
| --- | --- | --- | --- | --- |
| CLP + PBS | 20 | 9 (45%) | 8 | 6 |
| CLP + trans-CLP | 20 | 8 (40%) | 8 | 6 |
| CLP + trans-AKK | 20 | 12 (60%) | 8 | 6 |

Note: All enrolled mice were included in the 7-day survival analysis. The larger initial sample size accounts for expected perioperative mortality after CLP. After the survival window, surviving mice that completed the full behavioral battery were used for behavioral testing (n = 8 per group); for terminal histological and molecular assays, a prespecified n = 6 mice per group was randomly selected from these survivors. Selection was based only on survival to the endpoint, completion of all behavioral tests, and adequate tissue quality after transcardial perfusion; no outcome-based selection was applied, and all exclusions were made before data unblinding.
